# Characterisation of the Corticospinal Tract Using Diffusion Magnetic Resonance Imaging in Unilateral and Bilateral Cerebral Palsy Patients

**DOI:** 10.21315/mjms2018.25.5.7

**Published:** 2018-10-30

**Authors:** Safwan Samsir, Rahimah Zakaria, Salmi Abdul Razak, Mohamed Saat Ismail, Mohd Zulkifli Abdul Rahim, Chia-Shu Lin, Nik Mohammad Faez Nik Osman, Mohammad Afiq Asri, Asma Hayati Ahmad

**Affiliations:** 1 Faculty of Psychology & Education, Universiti Malaysia Sabah, Sabah, Malaysia; 2 Department of Physiology, School of Medical Sciences, Health Campus, Universiti Sains Malaysia, 16150 Kubang Kerian, Kelantan, Malaysia; 3 Department of Paediatrics, School of Medical Sciences, Health Campus, Universiti Sains Malaysia, 16150 Kubang Kerian, Kelantan, Malaysia; 4 School of Health Sciences, Health Campus, Universiti Sains Malaysia, 16150 Kubang Kerian, Kelantan, Malaysia; 5 Department of Dentistry, School of Dentistry, National Yang-Ming University, Taipei, Taiwan

**Keywords:** cerebral palsy, diffusion MRI, probabilistic tractography

## Abstract

**Background:**

Neuroimaging is increasingly used to locate the lesion that causes cerebral palsy (CP) and its extent in the brains of CP patients. Conventional structural magnetic resonance imaging (MRI) does not indicate the connectional pattern of white matter; however, with the help of diffusion MRI, fibre tracking of white matter can be done.

**Methods:**

We used diffusion MRI and probabilistic tractography to identify the putative white matter connectivity in the brains of 10 CP patients. We tracked the corticospinal tract (CST) of the patients’ upper and lower limbs and calculated the white matter connectivity, as indexed by streamlines representing the probability of connection of the CST.

**Results:**

Our results show that diffusion MRI with probabilistic tractography, while having some relation with the clinical diagnosis of CP, reveals a high degree of individual variation in the streamlines representing the CST for upper and lower limbs.

**Conclusion:**

Diffusion MRI with probabilistic tractography provides the state of connectivity from lesioned areas to other parts of the brain and is potentially beneficial to be used as an adjunct to the clinical management of CP, providing a means to monitor intervention outcomes.

## Introduction

The variable clinical manifestations of cerebral palsy (CP) patients make it imperative for an accurate diagnosis in order to set rehabilitative management strategies and predict outcomes. In paediatric CP patients, this is particularly difficult due to poor functional capacities and cooperation during physical examination, which consequently affect treatment and prognosis (1). While a majority of CP patients have structural brain abnormalities present when scanned with conventional structural magnetic resonance imaging (MRI), some do not manifest any abnormalities (2). This may be because the insult is not grossly anatomic but is more at the connectional level, hence the need for a more effective imaging technique that is capable of capturing subtle abnormalities, especially lesions involving structural connectivity.

Diffusion MRI is an MRI-based imaging technique that evaluates the integrity and orientation of white matter fibres (3) by measuring the three-dimensional shape and direction of water molecule diffusion within the brain voxel by voxel (4, 5), thus providing anatomic information about the status of white matter structures that cannot be assessed by a conventional structural MRI (6). Water molecules diffuse more freely within cerebral white matter in the direction of axonal fascicles rather than across them, due to the restriction of free water diffusion by the axonal membrane, axonal microtubules and myelin sheath (7, 8). Therefore, quantification of the orientation preference of diffusion may be related to axonal orientations (9). Such directional dependence of diffusivity is termed anisotropy (10). This technique has been increasingly used to investigate and gather qualitative and quantitative information regarding the microstructure of white matter in the last decade (11, 12).

Probabilistic tractography is a method that allows reconstruction of fibre tracts within the brain based on water diffusivity (12). Quantitative analysis using diffusion MRI tractography provides an objective measure of the white matter’s putative connectivity strength. The term ‘connection’ strictly means the probability of finding a tract connecting the seed to target region(s). While connection probability does not provide the number of axon bundles, its output is indicative of connection strength (13). In general, the number of streamlines in a probabilistic tractogram is a representation of the reproducibility of the tractogram, rather than the accuracy of the tractogram in representing the underlying neuroanatomy (14, 15).

In CP, injuries to the corticospinal tract and somatosensory thalamic radiations usually occur during the developmental stage of the brain (16). Injury to the upper motor neurons may decrease cortical input to the reticulospinal and corticospinal tracts (CST) and decrease motor unit effectiveness and motor control, causing muscle weakness and abnormal muscle control (17). Abnormalities of muscle tone, spasticity and motor abilities are closely related to the loss of integrity of the CST. Studies using diffusion tensor imaging (DTI) have demonstrated lower DTI parameters in the white matter tracts in patients with CP (18). However, few have focused on investigating quantitative measures of connectivity (19).

We aimed to investigate the association between probabilistic diffusion MRI tractography in CP patients and the type of CP based on clinical diagnosis. The findings may be used as an additional tool for assessing the individual variation in white matter tract deficiency to assist in the diagnosis and management of CP patients.

## Methods

### Participants

We recruited 10 spastic CP patients ranging from 8 to 18 years old. The patients had been diagnosed with CP and had been on follow-up in the paediatric neurology clinic for at least 6 months. Other inclusion criteria were the presence of hand function and the basic ability to grasp objects; Gross Motor Function Classification System Expanded and Revised (GMFCS-E & R) at least Level 2^1^ (20); and the ability to ambulate independently without an assistive device. Patients with any genetic syndromes, a history of orthopaedic or neurosurgery involving insertion of metal implants, a marked intellectual disability, or those that had received botulinum toxin injections within the past 6 months were excluded from the study. Other exclusion criteria include patients with an active seizure disorder or those who have had seizures for the past 6 months prior to commencement of the study, and presence of contraindications to MRI scanning.

### Diffusion MRI

#### Acquisition

Acquisition of brain images was performed in the Radiology Department of Hospital Universiti Sains Malaysia. None of the patients had ever had an MRI scan done. Prior to scanning, the patients were not allowed to consume food five to six hours before sedation. Sedation was given by a medical officer from the Department of Paediatrics to each of the patients to minimise head motion during scanning. Initially, oral chloral hydrate (50–75 mg/kg/dose) was given. If the patients were still not sedated, intravenous midazolam (0.1 mg/kg/dose) or ketamine (1.0 mg/kg/ dose) was administered. Intravenous atropine (0.01 mg/kg/dose) was given to decrease secretion for those who were given ketamine. To ensure patients’ safety and minimise other complications post-sedation, they were temporarily admitted to the ward and placed on pulse oximetry and cardiac monitoring until discharged.

All scans were performed using the 3.0T Philips Achieva MRI scanner with a 32-channel SENSE head coil. Prior to scanning, a survey scan was done in a sagittal orientation. Diffusion-weighted images were acquired using single-shot echo-planar imaging with a navigator echo. The diffusion acquisition parameters were as follows: number of directions = 32, acquisition matrix 96 × 96, reconstructed matrix = 128 × 128, field of view = 221 mm × 221 mm, repetition time/echo time = 10,726/76 ms, SENSE factor = 2, EPI factor = 67, b = 1000, flip angle = 90 and thickness = 2.3 mm (voxel size 2.3 × 2.3 × 2.3) for each of the 32 non-collinear diffusion-sensitising gradients. A T1-weighted, high-resolution structural image (TR/TE/slice/FOV = 9 ms/4 ms/4 mm slices/240 × 240 mm) with voxel size 1 × 1 × 1 mm was also obtained for verification of the brain’s anatomical regions.

#### Pre-processing of diffusion imaging data

The acquired images in dicom (.dcm) format were converted to niftii (.nii.gz) using MRIConvert (downloaded from http://lcni.uoregon.edu/~jolinda/MRIConvert/). The diffusion images were then inspected manually for any noise or artefacts. No datasets were excluded. Non-brain tissue was removed using the Brain Extraction Tool (BET) in FMRIB Software Library (FSL; http://www.fmrib.ox.ac.uk/fsl/) (21, 22), and betted images were corrected for motion and eddy-current-related distortions (23) using FMRIB Diffusion Toolbox (FDT in FSL). Automated modelling of crossing fibres (24) was performed using bedpostX in FDT with the following parameters: number of fibre orientations per voxel = 2, weight = 1, burnin = 1,000 and number of jumps = 1,250. Bedpostx produces a set of files ready for tractography.

#### Drawing of regions of interest

**Figure 1:**
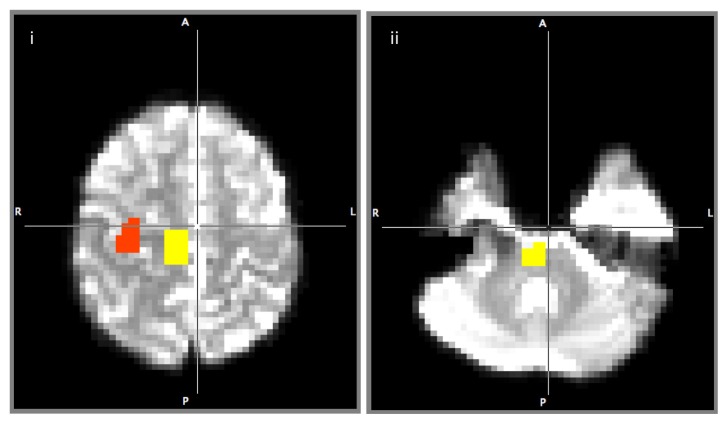
ROIs drawn on one subject’s diffusion image. Masks were hand-drawn on each subject’s brain image: (i) precentral knob (yellow), mediodorsal part of precentral gyrus (red) and (ii) lower pons (yellow)

The CST for both the upper and lower limbs were determined by a selection of fibres passing through regions of interest (ROI) designated as the seeds and target. Seed ROIs for the upper and lower extremities were according to known anatomy (ROI for the upper extremities: the precentral knob; ROI for the lower extremities: the mediodorsal part of M1 (primary motor cortex)) while the lower pontine area was used as the target ROI (25, 26, 27). Seed and target masks were hand-drawn in diffusion space (Figure 1) by a single investigator. Streamline fibre tracts passing through the ROIs were designated as the final tracts of interest.

### Probabilistic Tractography

**Figure 2:**
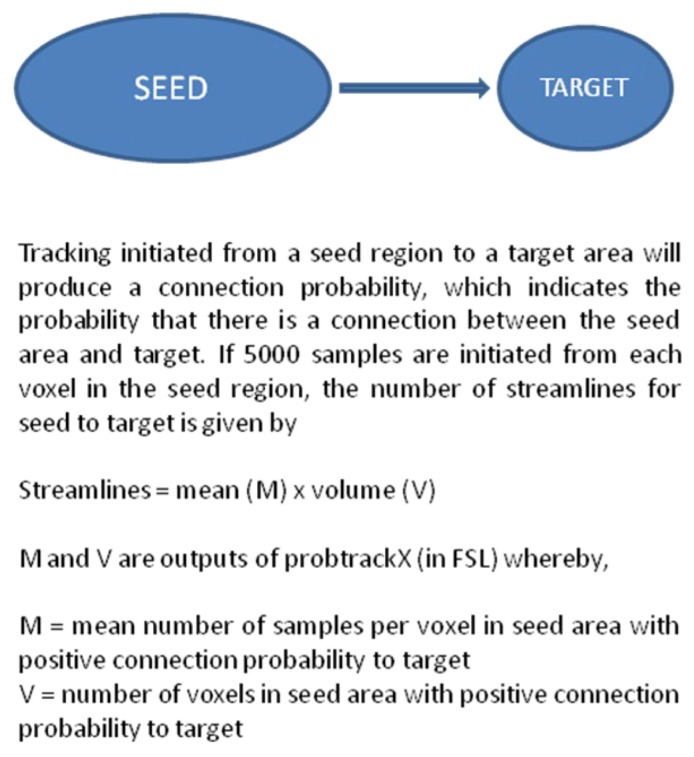
Calculation of number of streamlines

The probabilistic tractography algorithm used in this study was as described by Behrens et al. (28, 29). Probabilistic tractography was performed by probtrackx in FDT using 5,000 streamline samples. Streamline fibre tracts passing through both the seed and target ROIs were designated as the final tracts of interest. Quantitative analysis was performed by calculating the total number of streamlines between the seed and the target masks, derived from the multiplication of the number of voxels with positive connection probability by the mean number of positive connection probability (out of the 5,000 samples) per voxel as given by fslstats in FSL (calculation in Figure 2). The assumption is that the higher the number of streamlines, the stronger the white matter connectivity between the seed and target areas (30).

### Estimation of Effect Size of Connectional Reduction

To determine the reduction in connectivity between the lesioned side and non-lesioned side of CST in unilateral CP patients, the effect size was calculated using the following formula:

$$\begin{array}{r} \text{Reduction }(\%)=[(\text{streamlines on non}-\text{lesioned side}\\ -\text{streamlines on lesioned side})/\\ \text{streamlines on non}-\text{lesioned side}]\% \end{array}$$

## Results

### Descriptive Analysis

We recruited 10 spastic CP patients ranging from 8 to 18 years old (mean age ± SD = 12.7 ± 3.65, six males). Four of the patients had unilateral CP and six had bilateral CP (Table 1).

### Quantification of White Matter Connectivity of CST

In general, changes in patients with unilateral CP were more evident than those with bilateral CP with reduced streamlines on the side of the brain lesion, which more or less corresponded clinically with contralateral motor impairment. However, the degree of connectional strength implied varying involvement of the CST (Figures 3a–d). The effect size of the reduction in streamlines between the lesioned and non-lesioned side of the CST for each hemiparetic patient is shown in Table 2.

In patients with bilateral CP, the streamlines corresponding to the CST of both lower limbs were found to be bilaterally deficient compared to the streamlines representing the CST of the upper limbs. However, the patterns of connectivity did not always correspond with the clinical diagnosis. In patients with a clinical diagnosis of bilateral CP, the results showed that there were variabilities in the number of streamlines representing the CST for upper and lower limbs, indicating that the lesion was not confined to the white matter connections for lower limbs or upper limbs only (Figures 3e–j).

## Discussion

In general, our results showed a considerable degree of individual variation in the connectivity of the CST, as quantified by the probabilistic tractography that did not always correspond with the clinical picture.

The reduction in streamlines may exceed the clinical type of CP, and patients with a similar type of CP exhibit variable streamline patterns in the CST representing the upper and lower limbs on the lesioned side as well as the non-lesioned side of the brain. In our study, a patient clinically diagnosed as bilateral CP involving the lower limbs was seen to have decreased streamlines in the upper limbs as well, and a patient clinically having unilateral CP was also found to have considerable involvement of the ipsilateral CST in addition to the contralateral side. It is commonly known that patients may have occult lesions (e.g., bilateral lesions) despite a clinical diagnosis of unilateral CP (18). Another possible explanation is the occurrence of neuroplasticity in the brain, with non-lesioned areas taking over some of the functions of lesioned brain areas.

The state of white matter that forms the connections between brain areas which are crucial for brain function cannot be informed using conventional MRI, while diffusion tractography allows the imaging of white matter as represented by water diffusivity in the neuronal fibres. Probabilistic tractography is used to generate connection probability maps, which primarily show the reproducibility of connections between different brain areas rather than anatomical accuracy (14).

Most DTI studies on CP utilise other parameters such as fractional anisotropy and mean diffusivity to characterise white matter strength. For instance, Scheck et al. (19) found increased mean diffusivity between the anterior cingulate cortex and precuneus in the contralateral side of the brain of unilateral CP patients. Other studies found decreased fractional anisotropy in the transcallosal motor fibres (31), corticospinal tract (32), posterior limb of internal capsule (33, 34) and cerebral peduncles (34). Significant correlations have also been found between these parameters and severity of CP (35, 36, 34).

CP is not a progressive disease, and, per definition, the causing lesion has to be stationary and happened before 2 years of age. However, the clinical picture may be variable and changes in diffusion MRI parameters have been reported following intervention or training indicating plasticity of the CP brain. While CP is not known to progress or worsen, recent findings by Schertz et al. (37) showed that preintervention brain imaging predicts the benefit from bimanual intervention in patients with unilateral CP. Others have shown atypical activation patterns, including bilateral activation and activation in the ipsilateral hemisphere to the hand engaged in movement in unilateral CP (38, 39). Another benefit of quantitative probabilistic tractography is its feasibility to assess the effect of intervention in patients (13).

## Conclusion

Our findings show that probabilistic diffusion tractography in CP patients provides additional information regarding the state of white matter and the quantitative calculation of streamlines makes it appropriate to be used as an adjunct to clinical assessment in CP patients.

## Figures and Tables

**Figures 3a–d:**
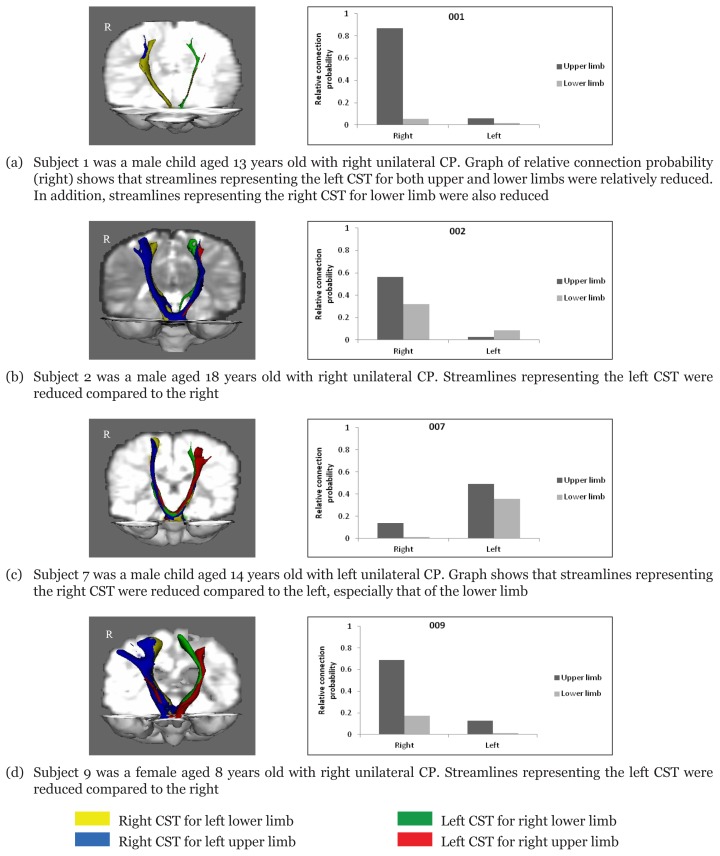
Diffusion images of the streamlines representing the corticospinal tract (CST) in children with unilateral CP and graph of relative connection probability calculated from each tract over the sum of all the four tracts. Tracts were seeded from the primary motor area to the target in the lower pontine area. Coloured tracts represent the CST corresponding to the motor pathways of the upper limbs (seeded from the precentral knob of the precentral gyrus) and lower limbs (seeded from the mediodorsal part of the precentral gyrus). Blue: right corticospinal tract for left upper limb; Yellow: right corticospinal tract for left lower limb; Red: left corticospinal tract for right upper limb; Green: left corticospinal tract for right lower limb

**Figures 3e–j:**
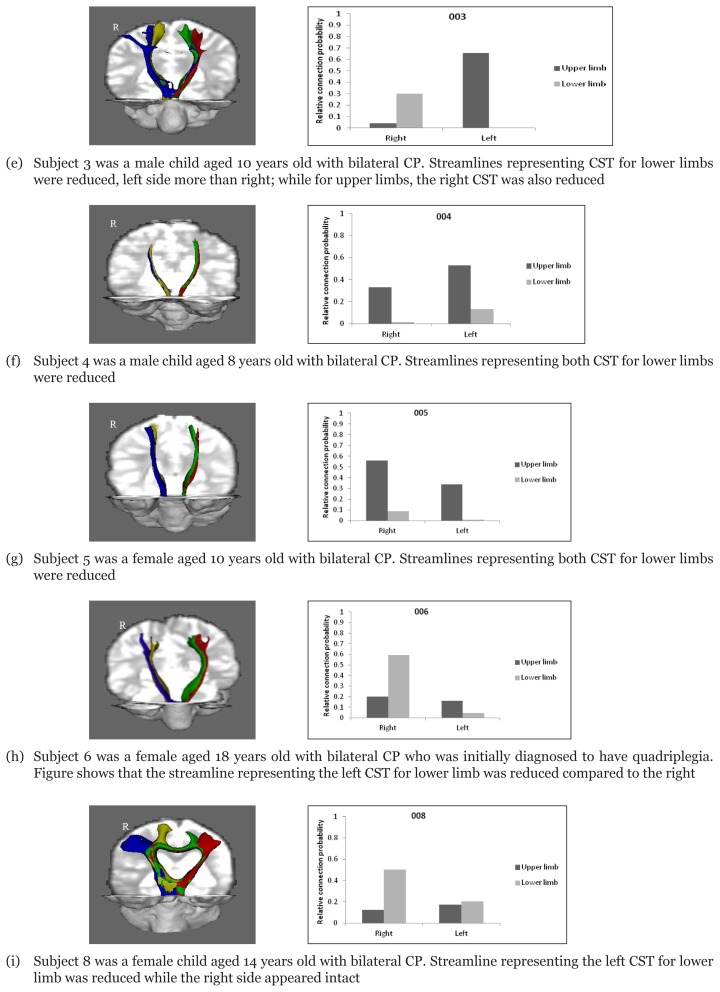

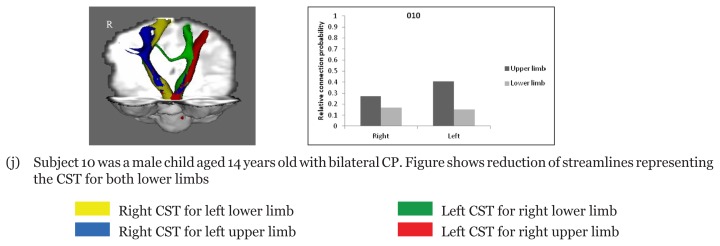
Diffusion images of the streamlines representing the corticospinal tract (CST) in children with bilateral CP and graph of relative connection probability calculated from each tract over the sum of all the four tracts. Tracts were seeded from the primary motor area to the target in the lower pontine area. Coloured tracts represent the CST corresponding to the motor pathways of the upper limbs (seeded from the precentral knob of the precentral gyrus) and lower limbs (seeded from the mediodorsal part of the precentral gyrus). Blue: right corticospinal tract for left upper limb; Yellow: right corticospinal tract for left lower limb; Red: left corticospinal tract for right upper limb; Green: left corticospinal tract for right lower limb.

**Table 1:** Descriptive data of participants

Participant	Age	Gender	Type of CP
1	13	Male	Unilateral
2	18	Male	Unilateral
3	10	Male	Bilateral
4	8	Male	Bilateral
5	10	Female	Bilateral
6	18	Female	Bilateral
7	14	Male	Unilateral
8	14	Female	Bilateral
9	8	Female	Unilateral
10	14	Male	Bilateral

**Table 2:** Effect size of reduction in connectivity index between lesioned and non-lesioned corticospinal tracts (CST) in unilateral cerebral palsy children

Participant	% Reduction in lesioned CST of upper limb	% Reduction in lesioned CST of lower limb
1	93.22	71.72
2	94.88	72.83
7	71.41	96.87
9	81.42	93.17

## Footnotes

1 Between 6th and 12th birthday: Children walk in most settings. Children may experience difficulty walking long distances and balancing on uneven terrain, inclines, in crowded areas, confined spaces or when carrying objects. Children walk up and down stairs holding onto a railing or with physical assistance if there is no railing. Outdoors and in the community, children may walk with physical assistance, a hand-held mobility device, or use wheeled mobility when traveling long distances. Children have at best only minimal ability to perform gross motor skills such as running and jumping. Limitations in performance of gross motor skills may necessitate adaptations to enable participation in physical activities and sports; Between 12th and 18th birthday: Youth walk in most settings. Environmental factors (such as uneven terrain, inclines, long distances, time demands, weather, and peer acceptability) and personal preference influence mobility choices. At school or work, youth may walk using a handheld mobility device for safety. Outdoors and in the community, youth may use wheeled mobility when traveling long distances. Youth walk up and down stairs holding a railing or with physical assistance if there is no railing. Limitations in performance of gross motor skills may necessitate adaptations to enable participation in physical activities and sports.
